# The French Observational Cohort of Usual Smokers (FOCUS) cohort: French smokers perceptions and attitudes towards smoking cessation

**DOI:** 10.1186/1471-2458-10-100

**Published:** 2010-02-26

**Authors:** Henri-Jean Aubin, Gérard Peiffer, Anne Stoebner-Delbarre, Eric Vicaut, Yasmine Jeanpetit, Anne Solesse, Geneviève Bonnelye, Daniel Thomas

**Affiliations:** 1 Hôpital Paul Brousse (AP-HP), Villejuif, France, Hôpital Emile Roux(AP-HP), Limeil-Brévannes, INSEM U699, France; 2 Pneumologie CHR METZ, France; 3 Epidaure Département de prévention CRLC Montpellier, France; 4 Unité de Recherche Clinique, AP-HP, Hôpital Fernand Widal, Paris, France; 5 PFIZER Paris, France; 6 TNS Healthcare, Montrouge, France; 7 Département de Cardiologie Médicale Institut de Cardiologie Groupe Hospitalier Pitié-Salpêtrière, Paris, France

## Abstract

**Background:**

Despite increasing governmental anti-smoking measures, smoking prevalence remains at a high level in France.

**Methods:**

The objectives of this panel study were (1) to estimate smoking prevalence in France, (2) to identify smokers' profiles according to their perceptions, attitudes and behaviour in relation to smoking cessation, (3) to determine predictive factors of quit attempts, and (4) to assess tobacco-related behaviours and their evolutions according to the changes in the smokers' environments. A representative sample of French population was defined using the quota method. The identified cohort of smokers was assessed, in terms of smoking behaviour, previous quit attempts, and intention to quit smoking.

**Results:**

A response rate of 66% for the screening enabled to identify a representative sample of the French population (N = 3 889) comprising 809 current smokers (21%). A majority of current smokers (63%) had made an attempt to quit smoking. Main reasons for having made the last attempt were cost (44%), social pressure (39%), wish to improve physical fitness (36%), fear of a future smoking-related disease (24%), and weariness of smoking (21%). Few attempts (16%) were encouraged by a physician. In those who used some kind of support (38%), NRT was the mostly used. Relapse was triggered by craving (45%), anxiety/stress (34%), a significant life event (21), weight gain (18%), and irritability (16%). Depression was rarely quoted (5%). Forty percent of smokers declared they intended to quit smoking permanently. Main reasons were cost (65%), physical fitness improvement (53%), fear of a future smoking-related disease (43%), weariness of tobacco (34%), and social pressure (30%). Using a smoking cessation treatment was considered by 43% of smokers that intended to quit. Barriers to smoking cessation were mainly fear of increased stress (62%), irritability (51%), and anxiety (42%), enjoying smoking (41%), and weight concerns (33%).

**Conclusion:**

Smoking prevalence and smoking cessation attempts rate were lower in this survey than in previous reports. Cost and social pressure were the main reasons for quitting smoking, maybe an effect of dramatic tax increases and smoking ban.

## Background

Total tobacco-attributable deaths are projected to rise from 5.4 million in 2005 to 6.4 million in 2015 and 8.3 million in 2030, being responsible for 10% of all deaths globally[1]. Populationwide-interventions that can reduce smoking prevalence are important for curbing the pandemic of tobacco-related disease. Different modalities provide evidence of effectiveness [2]: educational strategies, regulation of advertising and promotion, clean air regulations and restriction of minors' access to tobacco products, taxation on tobacco products, and treatment of nicotine addiction. In a recent comparison of 36 countries for tobacco dependence treatment services, France ranked third on a tobacco control scale, behind England and Scotland [3]. This encouraging score was based on the existence of an official treatment policy, an officially identified person managing treatment service, a national quitline, a specialized and easily available treatment system with a network of tobacco treatment specialists[4], medications availability, and a package reimbursement.

In France, tobacco use is estimated to cause every year about 60,000 premature deaths [5,6]. Smoking prevalence is estimated to be 29.9% [6], 31% [7], or 34.2% [8], depending on the survey. The ITC Four-Country survey has shown that at least 36% of smokers make a quit attempt in a given year [9]. Even though one can anticipate similar numbers in France, we haven't found spontaneous quitting attempt rates from French reports.

Tobacco control has become a priority for the French government, who strengthened its anti-tobacco policy in the early 2000s[7]. Cigarette taxes have dramatically increased in 2003, and again in 2004. The leading smoking cessation drugs have been made available in France: nicotine replacement therapy (1985), bupropion (2001), and varenicline (2007). The reported survey took place a few months before the implementation of several concomitant changes in tobacco control in France, all in February 2007: the smoking ban in public indoors, a financial support of up to 50 Euros a year per quitter by National Health Services, and the launch of the new smoking cessation drug Varenicline. Little is still known about smokers' behaviours in reaction to all these measures

The objectives of this population-based epidemiological panel study were:

• To estimate smoking prevalence in France;

• To identify smokers' profiles according to their perceptions, attitudes and behaviour in relation to smoking cessation;

• To determine predictive factors of quit attempts;

• To assess tobacco-related behaviours and their evolutions according to the changes in the smokers' environments.

This paper will present the study methodology and the main results obtained in the recruitment phase.

## Methods

This study has been carried out by the Market Research Company TNS Healthcare Sofres. Although no nominative data were recorded, the study was notified to the French personal data surveillance authorities (Commission Nationale Informatique et Libertés) and was conducted according to the relevant national and European laws and consensus professional guidelines. Subjects enrolled into the study were informed of the objectives of the study, the nature of transmitted data and their use, and their right to refuse.

### Study design

**Figure 1:**
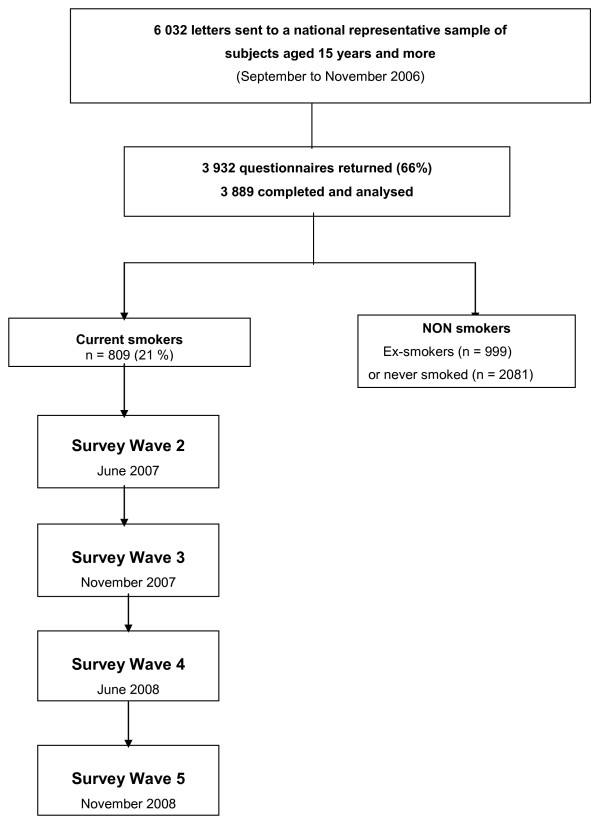
**Study Design**.

This survey is to be performed in five waves, several months apart, for a total follow up of 24 months. The study design is shown in Figure 1.

Wave 1 consisted in screening to select a national representative sample of smokers. A self-administered questionnaire was sent by mail to a national representative sample of 6,032 subjects aged 15 years or more from September 28th to November 13th 2006.

The four further waves were performed by phone (CATI system - Computer Assisted Telephone Interviewing). Data collection was performed by means of a questionnaire presented by TNS Sofres research interviewers. Smokers identified in Wave 1-screening were called in June 2007 (Wave 2), then in November 2007 (Wave 3), June 2008 (Wave 4), and finally November 2008 (Wave 5).

### Subjects

The screening was conducted among the ACCESS SANTE permanent polling base, representative of the French population. Its representativeness and algorithms of sample selection are permanently verified by experts from the Institut National de la Statistique et des Etudes Economiques (INSEE) and EUROSTAT, and regular audits are performed. Individuals entered in this base are recruited in several ways (face-to-face interviews, phone calls or mailings), thus reducing the possible risk of selection bias which could be associated with a particular methodology. Panellists participate in six to eight health surveys in a year and receive incentive services in return for their participation (no payment). The overall response rate is 70-75% for health surveys.

A self-administered questionnaire was mailed to a selected representative sample of 6,032 panellists aged 15 years and more, by using the quota method.

### Survey questionnaire

Apart from socio-demographic information, the screening questionnaire comprised 35 questions. The first question was aimed at establishing the smoking status: current smoker, ex-smoker, or never-smoker. For all participants, sociodemographics, risk factors and comorbidities were explored. For working subjects, distinction was made between high and low qualification.

For current smokers, questions were asked on smoking habits: daily or occasional use, number of daily cigarettes, duration of smoking, and age of first cigarette. The following conversion was established for non cigarette tobacco products: 1 cigarillo = 2 cigarettes; 1 cigar = 4 cigarettes; 1 pipe = 2 cigarettes. Level of dependence was evaluated by Fagerström Test for Nicotine Dependence (FTND)[10].

Information was collected on any attempt to quit smoking: number of attempts and time of first attempt. The last attempt was more thoroughly described: duration, role of health professionals, reasons which triggered the attempt, treatments used.

Last, the intention for a future smoking cessation was explored: any contemplation of smoking cessation, estimated timeline to attempted cessation, reasons for stopping, planned smoking cessation treatments, and factors which would influence the decision to stop. An adaptation of the "Barriers to smoking cessation checklist" was used [11].

Initially, the self-administered questionnaire had been tested on 8 smokers by means of face-to-face interviews in order to ensure a good comprehension.

### Statistical analysis

The representativeness of the sample was assessed by using the quota method[12], and computerised weighting was used, according to the Ranking Adjusted Statistics method [13], In order to reduce the bias due to non respondents to the screening mail, sociodemographic characteristics of the respondents were adjusted to the structure of the French general population aged 15 years and more [14]. Computerised weighting was therefore used on five criteria: gender (2 categories), age (5 categories), socioprofessional status (8 categories), region (9 categories), and community size (5 categories). Weighting the sample ensured it was still representative of the national population. The underlying reason is that only a sample with the same structure as the mother population on defined criteria allows generalising the answers brought to other criteria to the total population. A descriptive analysis was performed for each wave. All analyses were conducted using SAS software, version 8.0 (SAS Institute).

## Results

Out of 6,032 sent questionnaires, 3,932 were returned, i.e. the response rate was 66%. Of these, 43 (1%) were excluded because they were returned incomplete, and 3,889 were kept for analysis. As older and retired people were over-represented, weighting was comprised between 0.4 and 3.4, and its efficacy was 76.5%. After weighting, the sample of 3 889 individuals aged 15 years and more, was divided into 809 (21%) current smokers, 999 (26%) ex-smokers, and 2,081 (53%) never-smokers. Thus, extrapolation to the general population of France aged 15 years and more showed a smoking prevalence of 21%, i.e. approximately 10 million individuals. In order to have a better comparison of our results to a competing French survey [6,7], we also calculated the prevalence of smoking after exclusion of subjects over 75 years of age: the prevalence increased up to 22.1%.

Current smokers were predominantly young working males, with a rather low qualification. They had no increase of overweight/obesity or risk factor treatment prevalence compared to ex or never-smokers (Table 1).

**Table 1:** Sociodemographics and risk factors in current, ex, and never-smokers

	*Current smokers*	*Ex-smokers*	*Never-smokers*
N (%)	809 (21%)	999 (26%)	2081 (53%)
			
Male gender	56%	60%	39%
Age*	40.0 (15.0)	53.4 (16.4)	45.2 (19.9) A
Working	69%	54%	48%
Low qualification	47%	31%	24%
Body Mass Index (BMI)*	24.2 (4.5)	26.1 (4.7)	24.4 (4.5)
BMI ≥ 25 kg/m^2^	36%	54%	39%
Treated for a risk factor**	15%	36%	25%

* Mean (SD)

** Risk factors: hypertension, diabetes, hypercholesterolemia

### Smokers

Ninety percent smoked regularly (at least weekly), of whom 85% smoked everyday. The vast majority (95%) were cigarettes smokers, with a mean (SD) consumption of 14.2 (9.4) cigarettes a day. Median was at 13 cigarettes a day. Adding regular cigars, cigarillos and pipe smokers, the mean smoking rate reached 14.7 cigarette equivalents. Mean (SD) FTND score was 3.1 (2,6). Median score was 3.

In terms of duration, more than half the subjects (52%) have smoked for over 20 years, with a mean age of 16.5 years for the first cigarette (range: 5-50 years; median: 16 years).

A profile of smokers was established according to the fact they smoked more or less than 13 cigarettes per day (median taken as a cut off). Smokers above the median consumption (≥ 13 daily cigarettes) were more often males (61%), and they started smoking at a younger age (15.8 *vs *17.1 years-p < 0.01), but no difference was observed in terms of age category, work status or duration of smoking.

### Last attempt of smoking cessation

Sixty three percent of current smokers have made at least one attempt to stop smoking: 27% with one single attempt and 36% attempted several times. The chance of having made at least one attempt varied with age category: 46%, 70%, 64%, 62%, in age 15-24, 25-39, 40-49, and 50+ categories, respectively. Mean (SD) number of attempts was 2.2 (1.4) in smokers who made at least one attempt. Having made at least one attempt to quit smoking was significantly more frequent in employed rather than unemployed smokers (p < .01), and in daily than non daily smokers (p < .01). The median duration for the last attempt was 2 months, ranging from less than 24 hours (7%) to over 1 year (18%), the majority (56%) lasting 3 months or less.

Overall, 16% of last attempts to quit smoking were encouraged by a physician. They were mostly general practitioners (63%), then, far behind, cardiologists (18%) or gynecologists (14%). Chest physicians were only mentioned in 11% of cases, and tobacco treatment specialists in 9%. The majority of attempts was not encouraged by anybody in particular (Table 2). Family circles were particularly active. However the last attempt was mostly self-initiated.

**Table 2:** Persons encouraging the last attempt of smoking cessation.

	*Single attempt* *n = 219*	*Several attempts* *n = 287*
Physician (%)	9%	21%
Family circle (%)	35%	44%
Circle of friends (%)	6%	12%
Work colleagues (%)	3%	4%
Pharmacist (%)	1%	3%
Other (%)	1%	1%
Nobody in particular (%)	54%	46%
No answer (%)	4%	3%

The main reason for having made the last attempt to quit smoking was cost, mentioned by 44% of smokers, followed by social pressure (39%), the wish to improve physical fitness (36%), fear of a future smoking-related disease (24%), weariness of smoking (21%), and the existence of a pulmonary or cardiovascular condition (14%). Few smokers quoted smoking ban and other anti-smoking laws (4%), the marketing of a new smoking cessation drug (3%), or information campaigns (2%) as reasons to having made their last quit attempt.

Only 11% of smokers having made a quit attempt sought help from a physician when they attempted to stop smoking: 8% did so for their first and only attempt, and 13% when they made several attempts. Those who wished help from a physician mainly chose a general practitioner (77%); secondly tobacco treatment specialists were consulted (12%). Cardiologists (3%), chest physicians (2%), and gynecologists (2%) weren't often chosen. Mean visits to the physician was 2.2.

Even if support methods were more widely used by those who made several attempts (41% vs 25% for a first attempt, p < 0.01), still 62% did not use any support to quit smoking. Nicotine replacement therapy was mostly used: nicotine patch (20%), gums (12%), tablets (1%), and inhaler (1%). Bupropion was used in 5% of attempts. Other methods such as acupuncture, homeopathy, and hypnosis were also used as an aid to smoking cessation by 4% of the subjects.

Amongst the reasons that lead to resumed smoking, craving was the most frequently mentioned (45%), followed by anxiety/stress (34%), significant life event (21%), weight gain (18%), and irritability (16%). Other reasons were not reported more than in 5% of cases. Table 3 displays duration of attempts as a function of reason for relapse.

**Table 3:** Duration of attempts as a function of reasons for relapse.

*Reason of relapse*	*Frequency of report*	*Mean (SD) duration of attempts (months)*	*Median duration of attempts (months)*
Craving	45%	6.7(19.4)	.8
Anxiety/stress	34%	9(20.3)	1
Significant life event	21%	19.3(35.4)	6.2
Weight gain	18%	11.5(22.6)	4
Irritability	16%	3.8(9.7)	.4
Depression	5%	26.6(36.3)	12
Request of significant others	3%	1.4(1.6)	.8
Price of treatment	3%	2.7(2.9)	2
Treatment-emergent adverse events	3%	1.7(2.6)	.3

### Intention to quit smoking

Forty percent of current smokers declared they intended to stop permanently, more so in those who had previously made a quit attempt (52% vs 22%, p < .01). Of these, 5% intended to quit in the following week, 11% in the following 30 days, 55% in the following 6 months, 24% after 6 months, and 5% didn't specify. Intention to quit smoking was more frequent in daily than in non daily smokers (p < .01), but was unrelated to socio-demographic variables. Reasons mentioned comprised: high price of cigarettes (65%), wish to improve physical fitness (53%), fear of a future smoking-related disease (43%), weariness of tobacco (34%), social pressure (30%), and current smoking-related disease (14%). Other reasons mentioned were smoking ban in public places (8%), arrival of a new smoking cessation medication (7%), the feeling that smoking behavior is marginalizing (4%), and information campaigns by the media (2%). Forty-three percent of smokers who intended to quit definitively reported considering using a smoking cessation treatment. Nicotine patch was the most quoted treatment (18%), followed by nicotine gums (10%), bupropion (9%), varenicline - that was to be marketed several months after the survey - (5%), nicotine tablets (2%), and nicotine inhaler (1%). Other methods such as acupuncture, homeopathy, and hypnosis were quoted by 8% of the subjects.

Barriers to smoking cessation were mainly fear of increased stress, irritability, and anxiety, enjoying smoking, and weight concerns (table 4).

**Table 4:** Barriers to smoking cessation

*Barriers to smoking cessation*	*Yes*
Smoking helps me control stress	**62%**
Without cigarettes, I would feel too irritable to be around	**51%**
Without cigarettes, I would feel too anxious or worried about things	**42%**
I enjoy smoking too much to give it up	**41%**
It would be too hard to control my weight without smoking	**33%**
I don't know how to go about quitting smoking	**29%**
There are too many difficult things going on in my life right now	**28%**
I can't afford or find a smoking cessation program	**28%**
Smoking helps me control other behaviours that I have already changed	**17%**
I have tried to quit smoking in the past so many times, I've given up	**14%**
Without cigarettes, I would fell too down or sad	**14%**
My family and friends don't think it is important to quit smoking	**12%**

Questionnaire adapted from [11]

## Discussion

This survey found a surprisingly low smoking prevalence (21%) in the French population aged 15 or more. Of these, 85% were daily smokers. Thus, prevalence of daily smoking was 18%. Mean smoking rate was between 14.2 and 14.7 cigarettes per day, depending on the method used to convert non cigarette tobacco use. Prevalence of ex-smoking was 26%. Competing numbers come from the European survey on tobacco performed in 2008 in a representative sample of subjects aged 15 or more, showing a 34.2% smoking prevalence in France, a 28.3% daily smoking prevalence, and a 22.1% ex-smoking prevalence [8]. A French survey performed in 2005 in a representative sample of subjects aged 12-75 showed a smoking prevalence of 29.9%, a daily smoking prevalence of 24.9%, and an ex-smoking prevalence of 27.3% [6]. In this survey, mean smoking rate was 14.8 cigarettes per day in daily smokers, comparable to our results. Finally, a telephone survey performed in 2008 showed a smoking prevalence of 31% [7]. Why we found a lower smoking prevalence than others is unclear. To the least, our result suggests previously published smoking prevalence numbers [6-8] could be over-estimated.

Our results also show a lower rate of smoking cessation attempts (63%) in current smokers than in the 2005 national survey (76.5%) [6]. Yet, only one week success were taken into account in the 2005 survey, as our study considered all attempts, even of shorter duration. Other European surveys have shown smoking cessation attempts rate exceeding 70% [15]. Few of these attempts were prompted by physicians. This lack of medical involvement has been emphasized by others in France [6] and in other countries [16-19]. In our study, family circle seemed to have the lead in triggering smoking attempts, even though about half of the quit attempts weren't prompted by a third party. Unlike most studies [20-23] our survey didn't show that health concern was the primary reason for quitting smoking. Cost was the first reported reason for having made the last quit attempt or for a future attempt in our study. Dramatic increases of cigarette taxes in France in 2003 and 2004 have probably boosted this reason to quit in the lead position. Social pressure also ranked high in the reasons to quit smoking, reported second for the last attempt and forth for a future attempt. The idea of improving physical fitness was the most reported reason to quit smoking in the health concern category. Here again, this result differs from what is usually reported [20-22]. Information campaigns were not considered as a significant trigger of quit attempts in our study. Interestingly, the report of smoking ban as a reason to quit doubled when moving from last attempt to a future attempt. This survey took place several months before the introduction of the smoking ban in France (February 2007). It seems that some smokers anticipated the ban as a potential trigger for smoking cessation.

The relationship between reasons of relapse and duration of attempts is of some interest. Craving and some withdrawal symptoms (anxiety, irritability) tended to trigger the relapse quickly. Other withdrawal symptoms (weight gain and depression) triggered relapse much later. Anxiety, irritability, and depression are withdrawal symptoms that last 2-4 weeks [24]. In our study, not only depression was rarely reported, but it triggered relapse far later (median 12 months). Weight gain is considered more as an offset symptom [24], and indeed triggered delayed relapses in this study (median 4 months). Post-cessation weight gain was reported as a middle range barrier to smoking cessation (33%). French smokers, and particularly female smokers, are known to have a particularly high level of weight concerns [25].

As emphasized by other authors [26-29], a majority of smokers didn't use any form of smoking cessation medication during their last quit attempt. For those who used some form of support, nicotine patch was the most popular treatment. Price of treatment and treatment-emergent adverse events were rarely reported as reasons for relapse. It is noteworthy that, considering a future attempt, more smokers intend to use smoking cessation medications, even though 28% considered the price of a smoking cessation program as a significant barrier to smoking cessation.

Accordingly with the reported reasons for having relapsed from the previous quit attempt, this representative sample of smokers didn't seem to consider the risk of developing depression as a significant barrier to smoking cessation. In contrast, the risk of not controlling stress and of developing anxiety and irritability were the leading barriers to smoking cessation.

Finally, only 40% of smokers reported intending to quit smoking definitively. This may seem a small number compared to other reports [6,30]. However, most smokers declaring an intention to quit smoking, do not intend to do so definitively [31].

This study had some limitations: the response rate was 66%, and older and retired people were over-represented. The weighting however corrected this bias to some extent, since its efficacy was 76.5%. These sampling biases are in the range of French competing surveys [6,7].

## Conclusions

The most sticking findings from the analysis of the first wave of the FOCUS cohort are that smoking prevalence was much lower than in previous reports. Smoking cessation rates were also reduced. Reasons for these differences were unclear. Cost and social pressure were the main reasons for quitting smoking, maybe an effect of dramatic tax increases and smoking ban. Depression was not considered as a significant trigger for relapse or barrier to quit smoking.

## Competing interests

HJA has received sponsorship to attend scientific meetings, speaker honorariums and consultancy fees from Pfizer, McNeil, GlaxoSmithKline, Pierre-Fabre Sante, Sanofi-Aventis, and Merck-Lipha.

GP has received sponsorship to attend scientific meetings, speaker honorariums and consultancy fees from Pfizer, McNeil, GlaxoSmithKline, Pierre-Fabre Sante, Roche and Sanofi-Aventis

ASD has received sponsorship to attend scientific meetings from GlaxoSmithKline, Pierre-Fabre Sante, Pfizer, Sanofi-Aventis, McNeil, Novartis.

EV received lecture fees from the sponsor and honoraria for participation to advisory boards and steering committee meetings of another trial funded by Pfizer.

YJ and AS are employees of Pfizer.

The company of GB, TNS Healthcare, was funded by Pfizer.

DT has received sponsorship to attend scientific meetings, speaker honorariums and consultancy fees from Pfizer, speaker honorariums and consultancy fees from Pierre Fabre Santé, speaker honorariums from McNeill and Novartis SF.

## Authors' contributions

HJA, GP, ASD, EV, YJ, AS, GB, DT conceived and designed the study and participated to the discussion of the results, and helped top draft. HJA wrote the manuscript. GB performed the survey and the statistical analysis. YJ and AS coordinated the study. All authors read and approved the final manuscript.

## Pre-publication history

The pre-publication history for this paper can be accessed here:

http://www.biomedcentral.com/1471-2458/10/100/prepub
